# Diagnostic value of urinary tissue inhibitor of metalloproteinase-2 and insulin-like growth factor binding protein 7 for acute kidney injury: a meta-analysis

**DOI:** 10.1186/s13054-017-1660-y

**Published:** 2017-03-25

**Authors:** Hui-Miao Jia, Li-Feng Huang, Yue Zheng, Wen-Xiong Li

**Affiliations:** Department of Surgical Intensive Care Unit, Beijing Chao-yang Hospital, Capital Medical University, 8 Gongren Tiyuchang Nanlu, Chaoyang District, Beijing, 100020 China

**Keywords:** Tissue inhibitor of metalloproteinase-2, Insulin-like growth factor binding protein 7, Acute kidney injury, Diagnosis

## Abstract

**Background:**

Tissue inhibitor of metalloproteinase-2 (TIMP-2) and insulin-like growth factor binding protein 7 (IGFBP7), inducers of G_1_ cell cycle arrest, are two recently discovered good biomarkers for early diagnosis of acute kidney injury (AKI). To obtain a more robust performance measurement, the present meta-analysis was performed, pooling existing studies.

**Methods:**

Literature in the MEDLINE (via PubMed), Ovid, Embase, and Cochrane Library databases was systematically searched from inception to 12 October 2016. Studies that met the set inclusion and exclusion criteria were identified by two independent investigators. The diagnostic value of urinary [TIMP-2] × [IGFBP7] for AKI was evaluated by pooled sensitivity, specificity, likelihood ratio (LR), diagnostic odds ratio (DOR), and summary receiver operating characteristic (SROC) curve analyses. The causes of heterogeneity were explored by sensitivity and subgroup analyses.

**Results:**

A total of nine published and eligible studies assessing 1886 cases were included in this meta-analysis. Early diagnostic value of urinary [TIMP-2] × [IGFBP7] for AKI was assessed using a random-effects model. Pooled sensitivity and specificity with corresponding 95% CIs were 0.83 (95% CI 0.79–0.87, heterogeneity *I* ^*2*^ = 68.8%) and 0.55 (95% CI 0.52–0.57, *I* ^*2*^ = 92.9%), respectively. Pooled positive LR, negative LR, and DOR were 2.37 (95% CI 1.87–2.99, *I* ^*2*^ = 82.6%), 0.30 (95% CI 0.21–0.41, *I* ^*2*^ = 43.4%), and 9.92 (95% CI 6.09–16.18, *I* ^*2*^ = 38.5%), respectively. The AUC estimated by SROC was 0.846 (SE 0.027) with a Q* value of 0.777 (SE 0.026). Sensitivity analysis indicated that one study significantly affected the stability of pooled results. Subgroup analysis showed that population setting and AKI threshold were the key factors causing heterogeneity in pooled sensitivity and specificity.

**Conclusions:**

On the basis of recent evidence, urinary [TIMP-2] × [IGFBP7] is an effective predictive factor of AKI.

**Trial registration:**

PROSPERO registration number: CRD42016051186. Registered on 10 November 2016.

## Background

Acute kidney injury (AKI) is a common disorder of critically ill patients, especially in the intensive care unit (ICU), and a potential life-threatening factor closely associated with prolonged ICU stay, severe complications, and increased mortality. Prevention and identification of AKI in the early stage is important for improving the prognosis of critically ill patients [1, 2]. Although considered a standard tool in clinical routine tests, serum creatinine and urine output are not suitable for the early detection of AKI, owing to inherent methodological problems [3, 4]. Novel biomarkers for detecting AKI (i.e., neutrophil gelatinase-associated lipocalin [NGAL], kidney injury molecule-1 [KIM-1], and liver-type fatty acid-binding protein [L-FABP]) show earlier recognition of AKI [5–7]. Notably, urinary tissue inhibitor of metalloproteinase-2 (TIMP-2) and insulin-like growth factor-binding protein 7 (IGFBP7) were recently discovered and considered to be superior to NGAL, KIM-1, and L-FABP [8]. Both TIMP-2 and IGFBP7 are inducers of G_1_ cell cycle arrest, considered a key mechanism of AKI [9]. Kashani and colleagues [8] conducted the Stenting and Angioplasty with Protection in Patients at High Risk for Endarterectomy study to identify and validate novel biomarkers of AKI. The results showed that urinary [TIMP-2] × [IGFBP7] yields an AUC of 0.8 for predicting the development of AKI (Kidney Disease: Improving Global Outcomes [KDIGO] stage 2 or 3) within 12 h, indicating its superiority over 340 previously studied AKI proteins. Furthermore, other studies confirmed the good predictive performance of urinary [TIMP-2] × [IGFBP7] for AKI [10, 11]. Therefore, the present meta-analysis was performed to obtain a more robust performance measurement of [TIMP-2] × [IGFBP7] for early detection of AKI with more reliable evidence for clinical decision making.

## Methods

### Data sources and search strategy

A protocol of complete meta-analysis was constructed and adhered to the Preferred Reporting Items for Systematic Reviews and Meta-Analyses (PRISMA) standards [12]. The protocol was registered with the PROSPERO database (registration number CRD42016051186).

Two investigators (HMJ and LFH) independently searched the literature in the MEDLINE (via PubMed search engine), Ovid, Embase, and Cochrane Library electronic databases from inception to 12 October 2016. Text words or medical subject headings containing “tissue inhibitor of metalloproteinase-2,” “TIMP-2,” “insulin-like growth factor binding protein 7,” “IGFBP7,” “acute kidney injury,” “AKI,” “acute renal failure,” “ARF,” “acute kidney disease,” and “acute kidney stress” were researched without language restriction. Additional studies were identified by reviewing the reference lists of relevant articles.

### Study selection criteria

Two investigators (HMJ and LFH) independently screened the records obtained from the databases. Studies were included if they met the following three criteria:

1. Original clinical studies with participants over the age of 18 years (without restriction for study design)
2. Detection of urinary [TIMP-2] × [IGFBP7] used for early diagnosis of AKI
3. Development of AKI diagnosed by the criteria of risk, injury, failure, loss, end-stage kidney disease (RIFLE); the Acute Kidney Injury Network; or KDIGO [13–15]

Original human studies were excluded if they had insufficient information for true-positive (TP), false-positive (FP), false-negative (FN), and true-negative (TN) results. Repeated reports based on the same study data were also excluded. All retrieved articles were initially screened by title and abstract. The relevant ones were subsequently rescreened by full text. A third party resolved any discrepancies between two investigators (HMJ and LFH) in the process of study selection.

### Data extraction and quality assessment

Two investigators (HMJ and LFH) independently extracted study characteristics and data from individual reports, including first author, year of publication, study location, study design, population setting, AKI definition, sample size, sampling time of urine specimens, assay of urine TIMP-2 and IGFBP7, and numbers of AKI and non-AKI patients. Meanwhile, TP, FP, FN, TN, sensitivity, specificity, AUC, and optimal cutoff value of urinary [TIMP-2] × [IGFBP7] for early diagnosis of AKI were recorded. If multiple time points of urine measurements were used for AKI diagnosis, the one with the highest AUC was recorded. Similarly, if multiple cutoff values were obtained via AUC analysis, the one showing the highest Youden index was recorded.

Two investigators (HMJ and LFH) independently assessed the methodological quality of eligible studies using the Quality Assessment of Diagnostic Accuracy Studies 2 (QUADAS-2) tool [16]. QUADAS-2, focusing on risk of bias in the accuracy and applicability of original articles, consists of four domains: patient selection, index test, reference standard, and flow and timing. Each domain assesses the risk of bias by representative questions whose corresponding answers may be “yes,” “no,” or “unclear.” If all answers were “yes” in one domain, low risk of bias was considered. Any “no” in one domain indicated a high risk of bias. Studies not providing sufficient information to answer “yes” or “no” in the domain reflected unclear risk of bias. Methods used to assess the applicability of studies are the same as those of risk of bias. A third party resolved any discrepancies between two investigators (HMJ and LFH) in the process of data extraction and quality assessment.

### Statistical analysis

Meta-DiSc 1.4 software was used for statistical analysis. A random-effects model (DerSimonian and Laird method) or fixed-effects model (Mantel-Haenszel method) was constructed to estimate pooled sensitivity, specificity, positive likelihood ratio (PLR), negative likelihood ratio (NLR), and diagnostic odds ratio (DOR) with 95% CI. Model selection was based on the heterogeneity of included studies [17, 18]. Heterogeneity induced by threshold effect was reflected by a typical shape of a “shoulder-arm” in the summary receiver operating characteristic (SROC) plane or *p* < 0.05 in Spearman’s correlation coefficient test. Heterogeneity induced by a nonthreshold effect was evaluated by Cochran’s *Q* test and *I* ^2^ test; Cochran’s *Q* test with *p* < 0.10 indicated heterogeneity, whereas different *I* ^*2*^ values reflected low (<30%), moderate (30% to 50%), and high (>50%) degrees of heterogeneity. To identify the causes of heterogeneity in eligible studies, sensitivity and subgroup analyses were performed. The stability of results was examined by omitting one study at a time in sensitivity analysis; different subgroups based on potential sources of heterogeneity were considered in subgroup analysis. The SROC curve was used to estimate AUC [19], with a value ≥0.70 considered to reflect a useful predictive factor. Publication bias was assessed via funnel plot using Review Manager 5.3 software [20].

## Results

### Study selection and characteristics

**Fig. 1:**
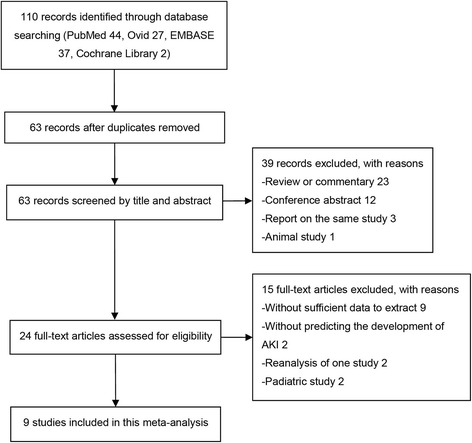
Study flow diagram. A diagram summarizing the search and screening process of the included studies. *AKI* Acute kidney injury

A flow diagram summarizing the study selection process is presented in Fig. 1. A total of 110 related reports were initially obtained from the databases, including 44 from PubMed, 27 from Ovid, 37 from Embase, and 2 from Cochrane Library. After removing duplicates, 63 articles were screened by title and abstract. Twenty-four hits were subsequently rescreened by full text after exclusion of 39 reports. Finally, 9 eligible studies assessing 1886 cases were included in this meta-analysis summarizing the predictive value of urinary [TIMP-2] × [IGFBP7] for AKI. The nine prospective cohort studies were published from 2013 to 2016, with sample sizes between 40 and 728. Most of them were conducted in the United States and Germany, with a cutoff value of 0.3 (ng/ml)^2^/1000. Notably, population settings were different in the nine studies, including postoperative cardiac patients, critically ill individuals in the ICU, and patients in the emergency department (ED) [7, 21–28]. The full KDIGO criteria were used to diagnose AKI in all nine studies, but AKI thresholds were different. Three studies defined primary clinical endpoint as patients meeting KDIGO stage 1 criteria. The remaining six studies considered the primary endpoint as patients meeting KDIGO stage 2 or 3 criteria. Urinary [TIMP-2] × [IGFBP7] was evaluated for AKI prediction within 12 h in four studies, within 48 h in four studies, and within 72 h in one study. The characteristics of individual studies are shown in Table 1.

**Table 1:** Characteristics of the included studies

First author, year [reference]	Location	Design	Setting	AKI threshold	Sample size (*n*)	AKI (*n*)	Non-AKI (*n*)	Sampling time	Assay
Kashani, 2013 [8]	North America and Europe	PC	ICU	KDIGO stage 2 or 3 within 12 h	728	101	617	At enrollment	NephroCheck test (Astute Medical, San Diego, CA, USA)
Bihorac, 2014 [21]	USA	PC	ICU	KDIGO stage 2 or 3 within 12 h	408	71	337	At enrollment	NephroCheck test
Hoste, 2014 [22]	USA	PC	ICU	KDIGO stage 2 or 3 within 12 h	153	27	126	At enrollment	NephroCheck test
Meersch, 2014 [23]	Germany	PC	CS with CPB	KDIGO stage 1 within 72 h	50	26	24	4 h after coming off CPB	NephroCheck test
Wetz, 2015 [24]	Germany	PC	CS with CPB	KDIGO stage 1 within 2 postoperative days	42	16	26	Day 1 after surgery	NephroCheck test
Pilarczyk, 2015 [25]	USA	PC	CABG	KDIGO stage 2 or 3 within 48 h	60	6	54	Day 1 after surgery	NephroCheck test
Gocze, 2015 [26]	Germany	PC	Surgery in ICU	KDIGO stage 1 within 48 h	107	45	62	At enrollment	NephroCheck test
Kimmel, 2016 [27]	Germany	PC	ED	KDIGO stage 2 or 3 within 12 h	298	46	252	Within 24 h	NephroCheck test
Dusse, 2016 [28]	Germany	PC	TAVI	KDIGO stage 2 or 3 within 48 h	40	8	32	Day 1 after surgery	NephroCheck test

*Abbreviations: AKI* Acute kidney injury, *ICU* Intensive care unit, *ED* Emergency department, *KDIGO* Kidney Disease: Improving Global Outcomes, *PC* Prospective cohort, *CS* Cardiac surgery, *CPB* Cardiopulmonary bypass, *CABG* Coronary artery bypass surgery, *TAVI* Transcatheter aortic valve implantation

### Quality assessment

**Fig. 2:**
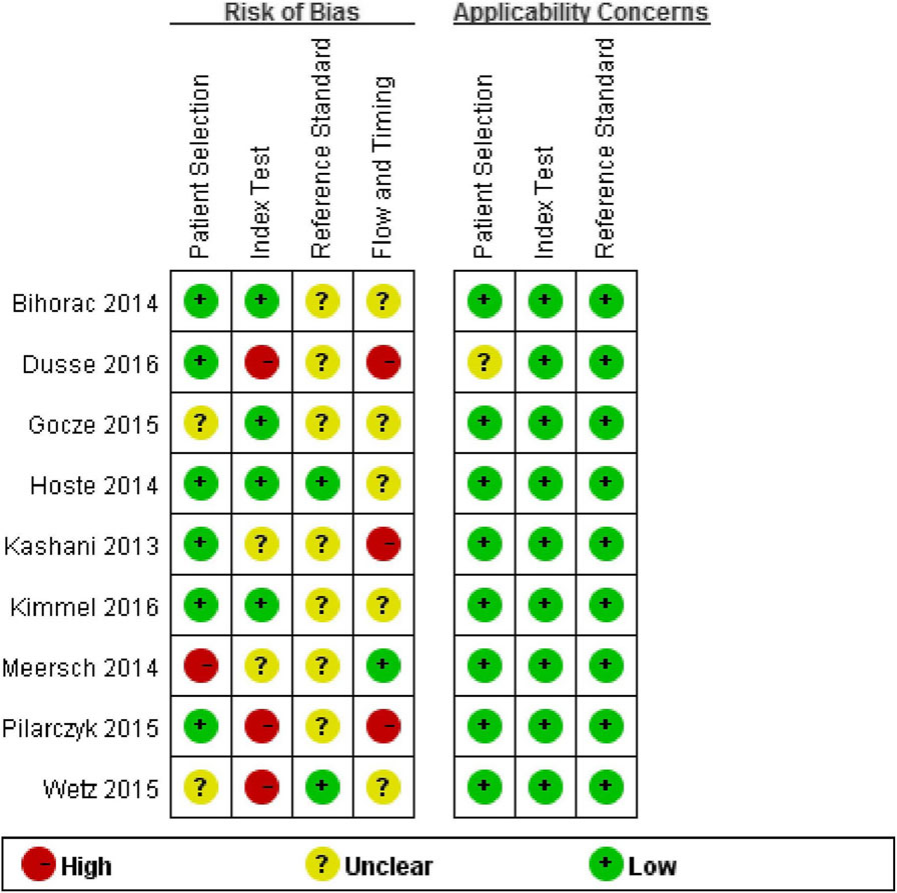
Quality assessment summary in each domain for individual studies. The quality assessment focusing on risk of bias and applicability concerns consists of four domains, including “patient selection,” “index test,” “reference standard,” and “flow and timing.” *Green*, *yellow*, and *red* indicate low, moderate, and high risk of bias, respectively

**Fig. 3:**
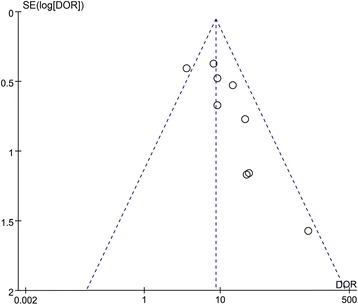
Funnel plot for the identification of potential publication bias in the included studies. *DOR* Diagnostic odds ratio

Study quality concerning each domain for individual studies is depicted in Fig. 2. Funnel plot (Fig. 3) results indicated a publication bias in the included studies.

### Data synthesis

**Fig. 4:**
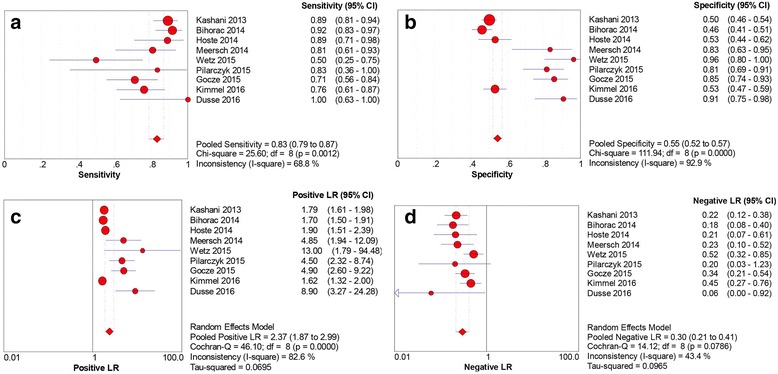
Sensitivity, specificity, positive likelihood ratio (LR) and negative LR of urinary [TIMP-2] × [IGFBP7] for predicting acute kidney injury in the nine studies. **a** Sensitivity. **b** Specificity. **c** Positive LR. **d** Negative LR. *IGFBP7* Insulin-like growth factor-binding protein 7, *TIMP-2* Tissue inhibitor of metalloproteinase-2

Data extracted from the nine eligible studies are summarized in Table 2. Studies showed different sensitivities, specificities, and AUC values of urinary [TIMP-2] × [IGFBP7] for early diagnosis of AKI. AUC values in these studies ranged from 0.71 to 0.97. Spearman’s correlation coefficient was 0.46 with *p* > 0.05, indicating there was no significant threshold effect in the nine studies. This result was also confirmed by the shape without a “shoulder-arm” in the SROC plane. In assessing the nonthreshold effect, pooled DOR (*I* ^*2*^ = 38.5%) and NLR (*I* ^*2*^ = 43.4%) showed moderate heterogeneity, whereas sensitivity (*I* ^*2*^ = 68.8%), specificity (*I* ^*2*^ = 92.9%), and PLR (*I* ^*2*^ = 82.6%) indicated significantly high heterogeneity. A random-effects model was used to pool the data. Pooled sensitivity and specificity were 0.83 (95% CI 0.79–0.87) and 0.55 (95% CI 0.52–0.57), respectively. Pooled PLR, NLR, and DOR were 2.37 (95% CI 1.87–2.99), 0.30 (95% CI 0.21–0.41), and 9.92 (95% CI 6.06–16.18), respectively. Estimated AUC was 0.846 (SE 0.027) with Q* of 0.777 (SE 0.026). The pooled sensitivity, specificity, PLR, and NLR for the nine studies are presented in Fig. 4.

**Table 2:** Diagnostic value of urinary [TIMP-2] × [IGFBP7] for acute kidney injury in individual studies

First author, year [reference]	Number of patients				Sensitivity (95% CI)	Specificity (95% CI)	AUC	Cutoff value
	TP	FP	FN	TP				
Kashani, 2013 [8]	90	313	11	314	0.89 (0.81–0.94)	0.50 (0.46–0.54)	0.80	0.3
Bihorac, 2014 [21]	65	182	6	155	0.92 (0.83–0.97)	0.46 (0.41–0.51)	0.82	0.3
Hoste, 2014 [22]	24	59	3	67	0.89 (0.71–0.98)	0.53 (0.44–0.62)	0.79	0.3
Meersch, 2014 [23]	21	4	5	20	0.81 (0.61–0.93)	0.83 (0.63–0.95)	0.81	0.3
Wetz, 2015 [24]	8	1	8	25	0.50 (0.25–0.75)	0.96 (0.80–1.00)	0.71	1.07
Pilarczyk, 2015 [25]	5	10	1	44	0.89 (0.36–1.00)	0.81 (0.69–0.91)	0.82	0.89
Gocze, 2015 [26]	32	9	13	53	0.71 (0.56–0.84)	0.85 (0.74–0.93)	0.85	0.3
Kimmel, 2016 [27]	35	118	11	134	0.76 (0.61–0.87)	0.53 (0.47–0.59)	0.76	0.3
Dusse, 2016 [28]	8	3	0	29	1.00 (0.63–1.00)	0.91 (0.75–0.98)	0.97	1.03

*Abbreviations: IGFBP7* Insulin-like growth factor-binding protein 7, *TIMP-2* Tissue inhibitor of metalloproteinase-2; *TP* True-positive, *FP* False-positive, *FN* False-negative, *TP* True-negative

**Fig. 5:**
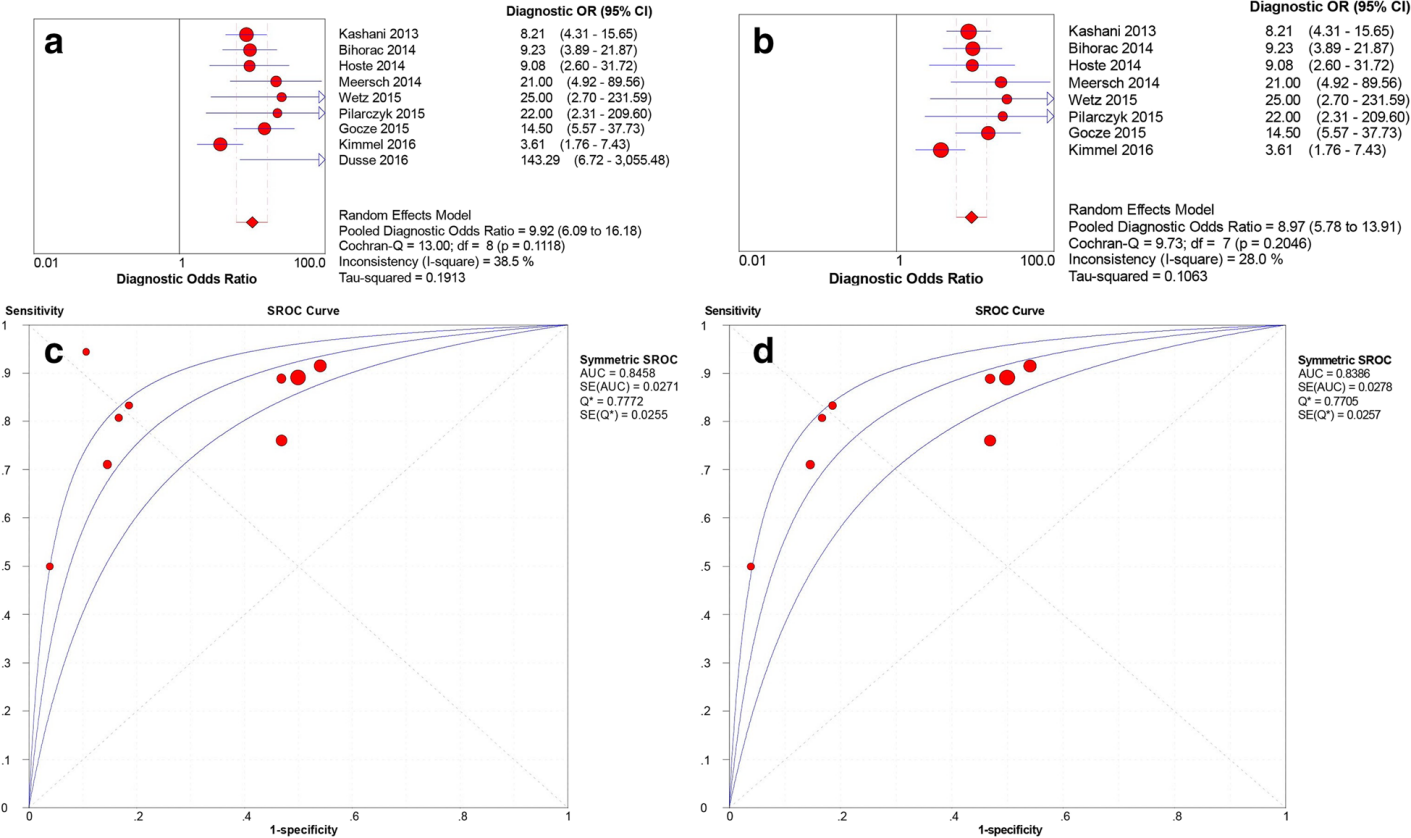
Pooled diagnostic OR (DOR) and summary receiver operating characteristic (SROC) curves of urinary [TIMP-2] × [IGFBP7] for acute kidney injury prediction. A comparison of pooled DOR and SROC curves between nine and eight studies. **a** Pooled DOR in nine studies. **b** Pooled DOR in eight studies. **c** SROC curve of nine studies. **d** SROC curve of eight studies. *IGFBP7* Insulin-like growth factor-binding protein 7, *TIMP-2* Tissue inhibitor of metalloproteinase-2

**Fig. 6:**
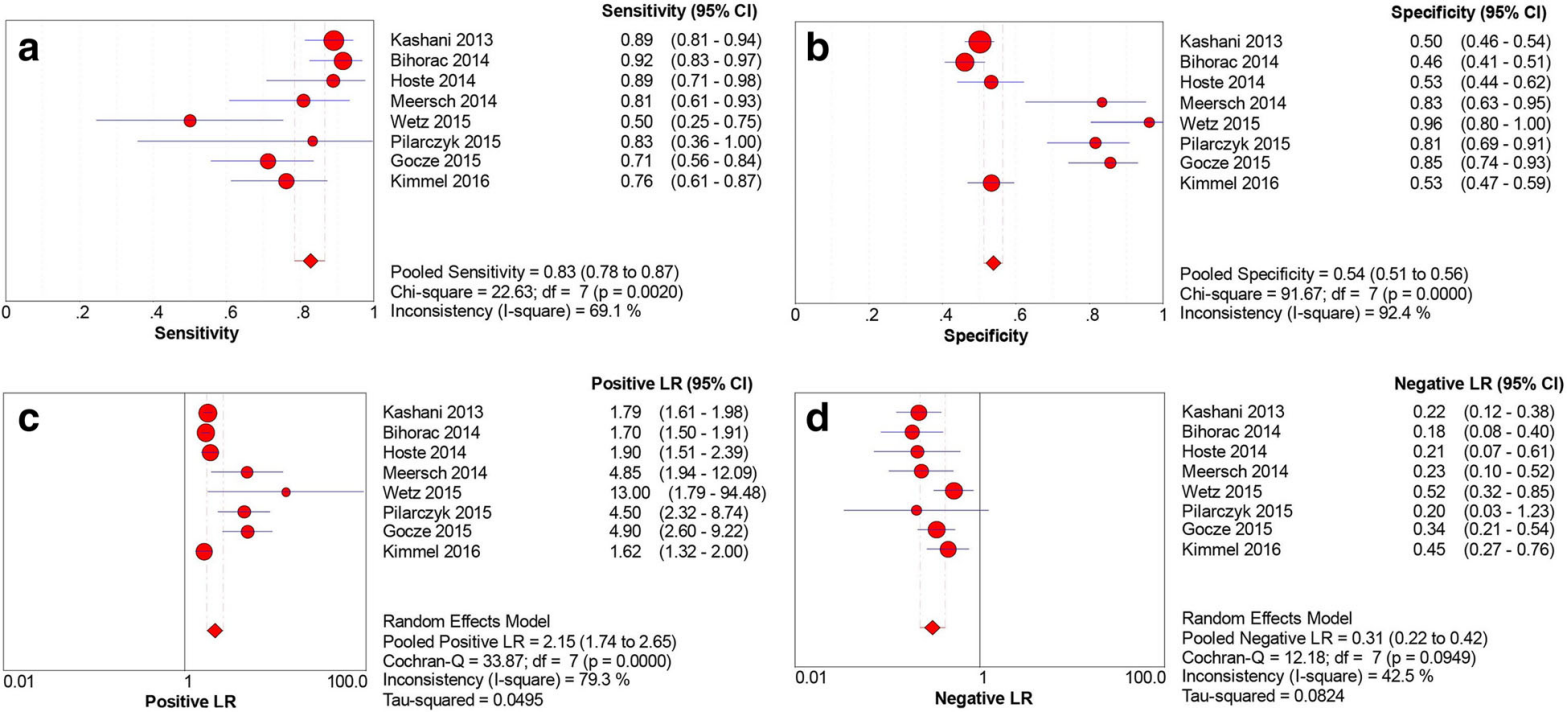
Sensitivity, specificity, positive likelihood ratio (LR) and negative LR of urinary [TIMP-2] × [IGFBP7] for acute kidney injury prediction in eight studies. **a** Sensitivity. **b** Specificity. **c** Positive LR. **d** Negative LR. *IGFBP7* Insulin-like growth factor-binding protein 7, *TIMP-2* Tissue inhibitor of metalloproteinase-2

In sensitivity analysis, the study by Dusse and colleagues [28] could affect the stability of pooled results. Omitting this study, the heterogeneity of pooled DOR decreased from moderate to low degree, with *I* ^*2*^ index decreasing from 38.5% to 28.0%. A slight reduction of *I* ^*2*^ was found in pooled specificity, PLR, and DLR. Pooled sensitivity, specificity, PLR, NLR, and DOR were 0.83 (95% CI 0.78–0.87, *I* ^*2*^ = 69.1%), 0.54 (95% CI 0.51–0.56, *I* ^*2*^ = 92.4%), 2.15 (95% CI 1.74–2.65, *I* ^*2*^ = 79.3%), 0.31 (95% CI 0.22–0.42, *I* ^*2*^ = 42.5%), and 8.97 (95% CI 5.78–13.91, *I* ^*2*^ = 28.0%), respectively. The estimated AUC was 0.839 (SE 0.028) with Q* of 0.771 (SE 0.026). A comparison of pooled DOR and SROC curves between eight and nine studies is shown in Fig. 5. Pooled sensitivity, specificity, PLR, and NLR for the eight studies are presented in Fig. 6.

Subgroup analysis was conducted based on population setting, AKI threshold, and blinding status. The results showed that population setting and AKI threshold were key factors causing heterogeneity. The 9 studies were divided into two subgroups according to different population settings. A total of 4 studies focused on patients undergoing cardiac surgery, and were termed the “cardiac surgery” subgroup. 4 others focused on patients in the ICU and 1 on patients in the ED, and constituted the “ICU and ED” subgroup. Interestingly, urinary [TIMP-2] × [IGFBP7] showed an excellent diagnostic value in patients undergoing cardiac surgery with estimated AUC of 0.911. Pooled sensitivity and specificity were 0.75 (95% CI 0.62-0.86, *I* ^*2*^ = 69.8%) and 0.87 (95% CI 0.80-0.92, *I* ^*2*^ = 33.9%), respectively. In the “ICU and ED” subgroup, estimated AUC was 0.814; pooled sensitivity and specificity were 0.85 (95% CI 0.80-0.89, *I* ^*2*^ = 68.5%) and 0.51 (95% CI 0.49-0.54, *I* ^*2*^ = 89.2%), respectively. Similarly, the 9 studies were divided into “KDIGO stage 2 or 3” and “KDIGO stage 1” subgroups according to the different AKI thresholds mentioned above. There were 6 studies in the “KDIGO stage 2 or 3” subgroup; estimated AUC was 0.813, while pooled sensitivity and specificity were 0.88 (95% CI 0.83-0.91, *I* ^*2*^ = 38.9%) and 0.52 (95% CI 0.49-0.55, *I* ^*2*^ = 89.8%), respectively. There were 3 studies in the “KDIGO stage 1” subgroup; estimated AUC was 0.868, with pooled sensitivity and specificity of 0.70 (95% CI 0.59-0.79, *I* ^*2*^ = 54.3%) and 0.88 (95% CI 0.80-0.93, *I* ^*2*^ = 31.7%), respectively. The results of subgroup analysis are shown in Table 3.

**Table 3:** Results of subgroup analysis based on different standards

Study		Sensitivity (95% CI)	Specificity (95% CI)	PLR (95% CI)	NLR (95% CI)	DOR (95% CI)	AUC
Nine studies		0.83 (0.79–0.87)	0.55 (0.52–57)	2.37 (1.87–2.99)	0.30 (0.21–0.41)	9.92 (6.09–16.18)	0.846
*I* ^2^ (%)		68.8%	92.9%	82.6%	43.4%	38.5%	
Population setting	Cardiac surgery (*n* = 4)	0.75 (0.62–0.86)	0.87 (0.80–0.92)	5.61 (3.54–8.89)	0.28 (0.12–0.67)	27.08 (9.87–74.34)	0.911
	*I* ^2^ (%)	69.8%	33.9%	0.0%	59.7%	0.0%	
	ICU and ED (*n* = 5)	0.85 (0.80–0.89)	0.51 (0.49–0.54)	1.83 (1.58–2.13)	0.29 (0.20–0.41)	7.66 (4.79–12.24)	0.814
	*I* ^2^ (%)	68.5%	89.2%	69.7%	35.7%	35.0%	
AKI threshold	KDIGO stage 2 or 3 (*n* = 6)	0.88 (0.83–0.91)	0.52 (0.49–0.55)	1.92 (1.62–2.29)	0.25 (0.17–0.39)	8.04 (4.49–14.40)	0.813
	*I* ^2^ (%)	38.9%	89.8%	74.3%	28.6%	43.1%	
	KDIGO stage 1 (*n* = 3)	0.70 (0.59–0.79)	0.88 (0.80–0.93)	5.20 (3.14–8.60)	0.37 (0.24–0.57)	17.04 (8.04–36.13)	0.868
	*I* ^2^ (%)	54.3%	31.7%	0.0%	42.0%	0.0%	
Blinding or not	Blinding (*n* = 6)	0.87 (0.82–0.91)	0.53 (0.51–0.56)	2.37 (1.83–3.08)	0.25 (0.18–0.34)	10.35 (6.83–15.70)	0.859
	*I* ^2^ (%)	58.7%	93.7%	84.8%	0.0%	0.0%	
	Not blinding (*n* = 3)	0.73 (0.62–0.82)	0.59 (0.53–0.65)	3.78 (1.00–14.23)	0.42 (0.28–0.63)	9.98 (2.41–41.41)	0.838
	*I* ^2^ (%)	59.0%	93.3%	84.9%	32.0%	68.4%	

*Abbreviations: AKI* Acute kidney injury, *ICU* Intensive care unit, *ED* Emergency department, *PLR* Positive likelihood ratio, *NLR* Negative likelihood ratio, *DOR* Diagnostic odds ratio, *KDIGO* Kidney Disease: Improving Global Outcomes

## Discussion

AKI remains one of the most common and serious clinical syndromes, and it is associated with high morbidity and mortality in critically ill patients. Current diagnosis of AKI is based on serum creatinine and urine output, despite known limitations of these markers. Diverse platforms and multiple studies have explored future biomarkers for early prediction of AKI [29]. In order to obtain a more robust performance measurement of urinary [TIMP-2] × [IGFBP7] for AKI, this meta-analysis was performed, also pooling existing studies. A total of 9 published and eligible studies assessing 1886 cases were included. The results indicated that urinary [TIMP-2] × [IGFBP7] is an effective predictive factor of AKI.

Dusse and colleagues demonstrated an excellent predictive value of urinary [TIMP-2] × [IGFBP7] for AKI in patients undergoing transcatheter aortic valve implantation (TAVI). An AUC of 0.97 was obtained, with sensitivity and specificity of 100% and 91%, respectively. However, sensitivity analysis showed that this study could result in significant heterogeneity in pooled DOR. Its small sample size and the specific population setting were considered important issues that can lead to heterogeneity. On one hand, the small sample size of 40 patients was likely to cause publication bias. On the other hand, patients undergoing TAVI are at high risk of developing AKI, which is probably associated with hypotension occurrence, reduced renal blood flow, and the use of contrast media during surgery [30, 31].

Subgroup analysis indicated that different population settings and AKI thresholds were the main sources of heterogeneity. Urinary [TIMP-2] × [IGFBP7] had a strong diagnostic value with an estimated AUC of 0.911 in the early stage of cardiac surgery-associated AKI. There are studies indicating that cardiac surgery with cardiopulmonary bypass could lead to reduced renal blood flow as well as surgical trauma, which probably damage kidney function or structure [32, 33]. Fortunately, with the development of innovative technologies such as renal Doppler ultrasound, renal blood flow and cortical microcirculation can be dynamically monitored at the bedside, which may help manage hemodynamics and decrease the incidence of ischemic AKI [34]. Further clinical trials are still required to confirm the diagnostic value of urinary [TIMP-2] × [IGFBP7] for AKI in patients undergoing noncardiac surgery.

The diagnostic value of urinary [TIMP-2] × [IGFBP7] was also influenced by different AKI thresholds. AUC was higher in the KDIGO stage 1 subgroup than in the KDIGO stage 2 or 3 subgroup (0.868 vs. 0.813). However, pooled sensitivity was lower in the KDIGO stage 1 (0.70) compared with that of the KDIGO stage 2 or 3 subgroup (0.88). The main causes may be that TIMP-2 and IGFBP7, reflecting the stress status of the kidney, are more relevant to kidney damage. AKI diagnosed by KDIGO stage 1 criteria, possibly with FP results, is affected by many factors of hemoconcentration, drugs, and reversible oliguria, which may not develop real kidney stress and damage. Although KDIGO stage 2 or 3 reflects a moderate to severe AKI, renal cells are more likely to sustain insults by sepsis and ischemia that cause kidney damage [35, 36]. On the basis of this hypothesis, urinary [TIMP-2] × [IGFBP7] may be more sensitive for predicting AKI with KDIGO stage 2 or 3 than AKI with KDIGO stage 1.

TIMP-2 and IGFBP7 are two biomarkers of G_1_ cell cycle arrest, indicating a preinjury status that leads to AKI [36]. AKI is related to the mechanisms of inflammation, oxidative stress, and apoptosis in cellular and molecular pathways [37, 38]. TIMP-2 and IGFBP7 can participate in these mechanisms and reflect early damage of the kidney [35]. They can regulate the activated p-protein cascade of p53, p21, and p27, subsequently blocking the effect of cyclin-dependent protein kinase complexes and altering the cellular response to the toxin or inflammatory factors [39–41]. Moreover, these two biomarkers may attempt to protect renal cells and avoid division, demise, or senescence [42, 43]. There is evidence showing that TIMP-2 and IGFBP7 are able to mark injured tubular epithelium and send signals in case of septic and ischemic insults, warning for kidney stress [44]. Then, renal tubular cells would enter for a short period G_1_ cell cycle arrest to prevent injury aggravation [45, 46]. Such physiopathological mechanisms and cellular pathways may help explain the use of these markers in early prediction of AKI. Further, the TOPAZ study recognized that combining urinary [TIMP-2] × [IGFBP7] with clinical factors could improve the predictive value in AKI compared with the biomarkers alone [21]. The AUC of combined urinary [TIMP-2] × [IGFBP7] and clinical information model was 0.86 (95% CI 0.80–0.90), higher than that of the clinical information model alone (AUC 0.70, 95% CI 0.63–0.76) and the urinary [TIMP-2] × [IGFBP7] test alone (AUC 0.82, 95% CI 0.76–0.88) [21]. These results suggested that a combination of clinical characteristics with specific biomarkers may enhance diagnostic accuracy in AKI. Like troponin, it constitutes an outstanding acute coronary syndrome biomarker when combined with typical symptoms and angina pectoris in specific populations. Similarly, there is renal angina (RA) in the AKI disorder. RA incorporates risk factors (e.g., advanced age, cardiopulmonary bypass, volume depletion, nephrotoxins) and symptomology (e.g., change of serum creatine, urine output, fluid overload) of patients to assess risk stratification for adult AKI. The renal angina index (RAI) is used for pediatric cohorts instead of RA to evaluate the risk for developing AKI by establishing point values [47, 48]. Clinical studies confirmed that a combined use of AKI biomarkers with RAI could improve the predictive value of the biomarkers for AKI [49]. Therefore, to a certain extent, this approach may be considerably potent for early prediction of AKI.

Serum creatinine and urine output were proposed by KDIGO for diagnosing AKI [15]. However, their use may inevitably result in inaccurate diagnosis of patients with AKI. Serum creatinine is readily available and specific for renal function. Nonetheless, it remains limited for AKI diagnosis. Serum creatinine can be affected by several factors, including age, diet, muscle mass, drugs, and creatinine’s volume of distribution. With a delayed reaction, serum creatinine concentrations take 24 to 36 h to rise after kidney damage. Urine output is far less specific and can be influenced by diuretics; it persists until renal function almost ceases. Severe AKI can present as anuria, oliguria, and normal urine output, and oliguria can occur in volume depletion without AKI [13, 50, 51]. Such problems may result in the limitation of using serum creatinine and urine output as the gold standard for diagnosing AKI. Furthermore, other limitations also existed in this meta-analysis. First, urinary [TIMP-2] × [IGFBP7] had a high predictive value for AKI in different population settings, but its predictive value was not identified in septic AKI, owing to limited studies. Second, with various sampling times in different studies, we did not explore the time window of urinary [TIMP-2] × [IGFBP7] for the diagnosis of AKI based on the same population. Third, we included some studies of small sample size, which could affect consistency in eligible studies and cause bias. Fourth, there was publication bias in the included studies; AUCs of urinary [TIMP-2] × [IGFBP7] for diagnosing AKI in these studies might be overestimated to a certain degree. Finally, the predictive value of urinary [TIMP-2] × [IGFBP7] for AKI progression and prognosis are also important issues in clinic. However, we did not directly explore these problems, owing to limited studies.

## Conclusions

On the basis of recent evidence, urinary [TIMP-2] × [IGFBP7] is an effective predictive factor of AKI. Further studies should determine how using this test can affect disease outcomes in the future.

## Key messages

- AKI, a common disorder in critically ill patients, is closely associated with prolonged ICU stay, severe complications, and increased mortality.
- Urinary [TIMP-2] × [IGFBP7] is an effective predictive factor of AKI, especially for patients undergoing cardiac surgery.
- A combined use of biomarkers with clinical characteristics of patients may help predict AKI in the future.

## Abbreviations

AKI: Acute kidney injury
CPB: Cardiopulmonary bypass
CS: Cardiac surgery
DOR: Diagnostic odds ratio
ED: Emergency department
FN: False-negative
FP: False-positive
ICU: Intensive care unit
IGFBP7: Insulin-like growth factor-binding protein 7
KDIGO: Kidney Disease: Improving Global Outcomes
KIM-1: Kidney injury molecule-1
L-FABP: Liver-type fatty acid-binding protein
NGAL: Neutrophil gelatinase-associated lipocalin
NLR: Negative likelihood ratio
PC: Prospective cohort
PLR: Positive likelihood ratio
PRISMA: Preferred Reporting Items for Systematic Reviews and Meta-Analyses
QUADAS-2: Quality Assessment of Diagnostic Accuracy Studies
RA: Renal angina
RAI: Renal angina index
RIFLE: Risk, injury, failure, loss, end-stage kidney disease
SROC: Summary receiver operating characteristic
TAVI: Transcatheter aortic valve implantation
TIMP-2: Tissue inhibitor of metalloproteinase-2
TP: True-negative
TP: True-positive
